# Lightweight design and static analysis of lattice compressor impeller

**DOI:** 10.1038/s41598-020-75330-z

**Published:** 2020-10-27

**Authors:** Yuan Zhang, Fanchun Li, Dejun Jia

**Affiliations:** Dalian Maritime University, 1 Linghai, Hightech Zone, Dalian, 116026 Liaoning People’s Republic of China

**Keywords:** Aerospace engineering, Mechanical engineering

## Abstract

Taking the compressor impeller as the research object and the lightweight design as the research goal, a lattice filled lattice cell suitable for the application of rotating periodic symmetric structure is designed. Its purpose is to make the rigidity and strength of impeller adjustable and reduce the mass of impeller on the premise of meeting the design requirements. The analysis and comparison of unfilled impeller, solid impeller and lattice filled impeller with different diameters were carried out under the limit condition of 80,000 r/min. The results showed that the average circumferential deformation of lattice impeller tip with beam diameter of 0.2 mm, 0.4 mm and 1 mm was 4.84%, 3.49% and 3.71% lower than that of solid impeller. For the impeller with a lattice beam diameter of 0.4 mm, its weight can be reduced by 22.68% compared with the solid impeller. The average circumferential deformation of the tip of the lattice impeller lies between the unfilled impeller and the solid impeller. The results show that the impeller with lattice filling hub can not only reduce the weight effectively, but also improve the efficiency of the compressor.

## Introduction

Lightweight design of structures is one of the important issues in the field of aerospace^1–3^. The application of lattice structure can realize the lightweight design of aircraft structure^4–6^. The application of lattice structure in the lightweight design of compressor impeller structure can not only reduce the overall structural mass of the aircraft, but also reduce the torque of the axle when the impeller starts and stops suddenly. The impeller axle structure can also be lightweight, and the aircraft structure mass will be further reduced. Therefore, under the premise of ensuring the structural safety and aerodynamic performance, metal lattice structure will have a very broad application prospect in the field of aerospace.

With the continuous progress of industrial manufacturing technology, in recent years, the design and processing of lattice structure has gradually matured. With the development of 3D printing technology and computer-aided design technology, engineers can design more complex structures which cannot be processed by traditional manufacturing. This can not only meet engineering requirements, but also have better performance. For example, bone implant structure in medicine^7,8^, aerospace structure based on topology optimization^9,10^, functional gradient lattice structure based on topology optimization^11–13^. This kind of structure can not only greatly reduce the weight of the structure, but also adjust the optimization direction according to the needs of the designer, so that the structure can reduce the weight and meet the needs of the specific requirement. For instance, Lin^11^ designs a kind of variable density lattice structure with elastic properties equivalent to the elastic properties of solid based on the topological optimization results by using the progressive homogenization method, which has a higher natural frequency than the original structure. When the lattice structure is applied to the lightweight design of the parts, the vibration and noise of the parts can be reduced while the structure weight is reduced, which is conducive to the absorption of collision energy^14^, and brings a safer and more comfortable environment for the users. With the continuous development of lattice technology and 3D printing technology, some researchers gradually apply lattice structure to engineering practice. Liao^15^ used the multi-scale topology optimization method based on the homogenization method to realize the variable density lattice topology optimization design for the connecting rod model. Stephen^16^ proposed a new lattice optimization method, which uses the principal strain field obtained from topology optimization to design the size and shape of each lattice structure in three-dimensional space, and designed a lattice support structure based on functional gradient. However, there are not many examples of applying lattice structure to engineering design. At present, most of them are based on the theoretical analysis and simple lattice structure design^17–19^. The research of applying lattice structure to lightweight design of impeller structure is still so few relatively.

Therefore, in our previous research^20^, a lattice based on conventional cubic lattice structure and rotating array lattice structure is proposed for the first time. Under the same load condition, the stress and deformation of rotating lattice filling and conventional lattice filling disk models are compared and verified, and the advantages and disadvantages of two lattice filling models are given in this paper. The rotating lattice structure (Hereinafter referred to as RL structure) is applied to the design of compressor impeller hub, and the rotating lattice filling impeller is printed by SLM Solutions Group AG Germany(SLM280)Metal 3D printer, which verifies its machinability. On this basis, the finite element analysis is carried out when only the rotating inertia load is considered, and compared with the analysis structure of solid impeller and unfilled impeller. The work of this paper will provide a new idea for the lightweight design of compressor impeller structure and a reference for the design and processing of lattice impeller.

## Result

**Figure 1:**
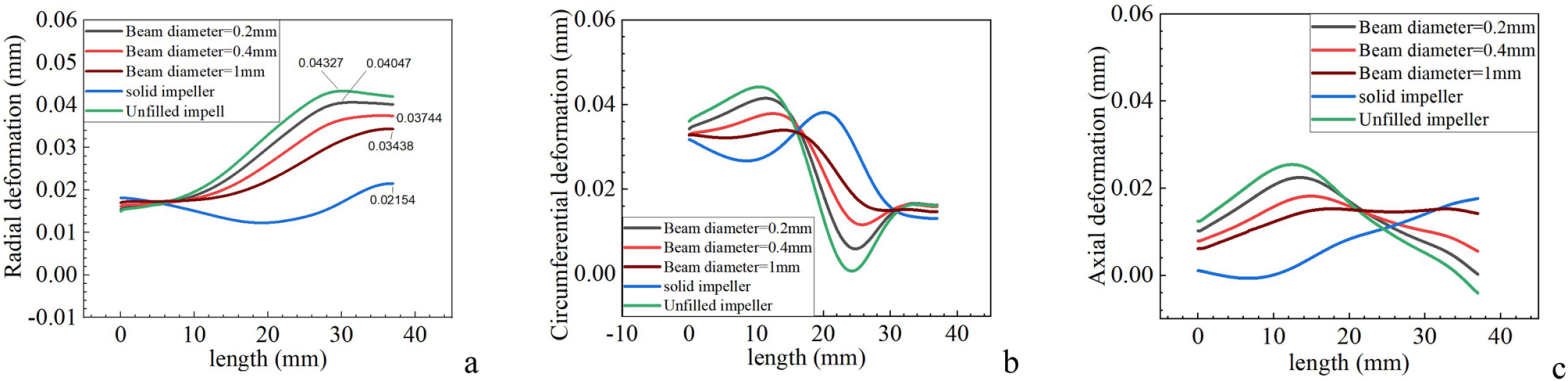
Deformation of path 1|| (**a**) Radial deformation; (**b**) Circumferential deformation; (**c**) Axial deformation.

In this section, the mechanical properties of lattice impellers with different lattice beam diameters are analyzed. The distribution of stress and deformation on path 1 and stress on path 2 were calculated for the rotating lattice impeller, unfilled impeller and solid impeller with the lattice beam diameter of 0.2 mm, 0.4 mm and 1 mm, respectively, under the condition of only considering the inertia load and rotating speed of 80,000 r/min. As the impeller is a rotating element, it is more meaningful to consider the deformation and stress distribution of the impeller in three directions under the cylindrical coordinate. For different impellers, the deformation distribution of path 1 in three directions is shown in Fig. 1.

When only considering the inertia load, the impeller is only affected by the centrifugal force outward along the radial direction at the stable speed, so the rotating impeller has the trend of outward expansion, and the extent of outward expansion is affected by the rigidity of the impeller. The stiffness performance of the lattice impeller must be between the solid impeller and the unfilled impeller, so the radial deformation of the lattice impeller should also be between the unfilled impeller and the solid impeller. The radial deformation distribution in Fig. 1a can better illustrate the rationality of the simulation results. The radial deformation of impeller will directly affect the shroud clearance of impeller. It can be seen from Fig. 1a that the maximum radial deformation of unfilled impeller is 0.04327 mm, while that of solid impeller is 0.02154 mm, and that of lattice impeller is between unfilled impeller and solid impeller. This means that the girth gap of lattice impeller will be smaller than that of solid impeller and larger than that of unfilled impeller. Therefore, in order to ensure the normal operation of the impeller, Therefore, in order to ensure the normal operation of the impeller, a larger shroud clearance is needed for the lattice impeller in static state compared with the solid impeller.

**Figure 2:**
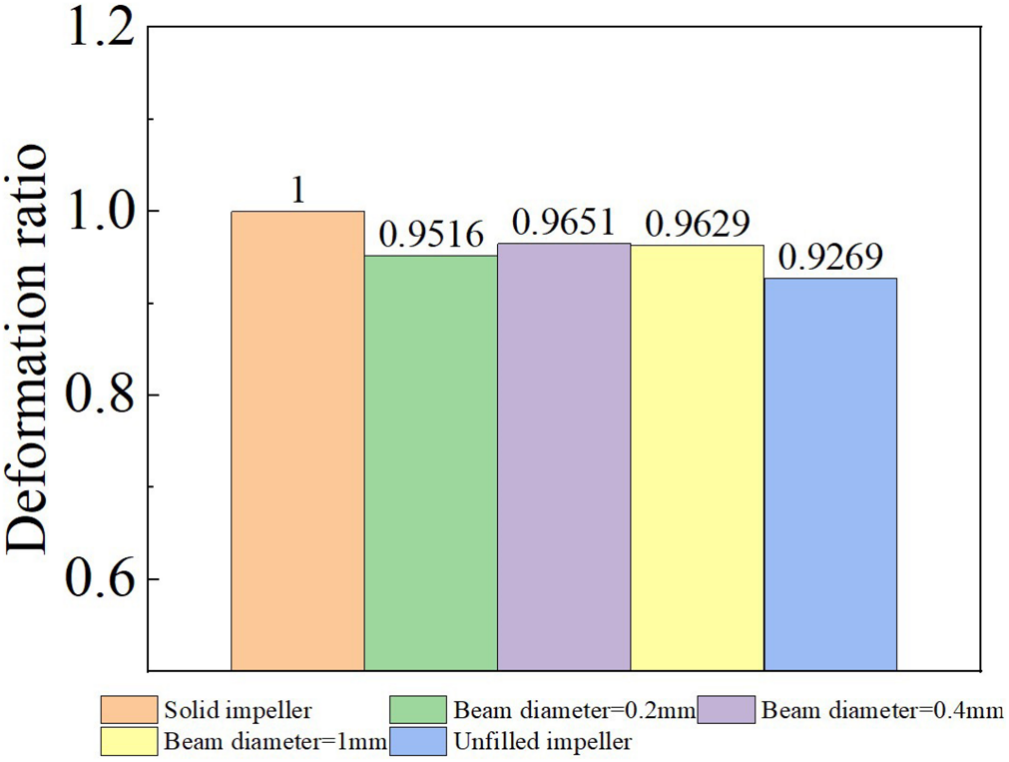
Average value of circumferential deformation.

For the circumferential deformation, it mainly affects the aerodynamic performance of the impeller structure, and the aerodynamic performance of the compressor impeller will directly affect the propulsion characteristics of the turbine engine. In practical design, we often assume the impeller structure as a rigid body for aerodynamic performance design. Therefore, the impeller with small circumferential deformation is the premise to ensure that the actual aerodynamic performance of the impeller is consistent with the design performance. Because this is a rotating structure, different mass makes the "centrifugal inertia force" of lattice impeller smaller than that of solid impeller and larger than that of unfilled impeller, which will affect the circumferential deformation of impeller. From Fig. 1b, we find that the circumferential deformation distribution of solid impeller is not always smaller than that of lattice impeller and solid impeller. For example, in the 0–15 mm segment, the deformation of solid impeller is smaller than that of other impellers, while in the 15–32 mm segment, the deformation of solid impeller is larger than others. To facilitate comparison, we take the average value of the circumferential deformation of all the collected data points on path 1. The ratio between the circumferential deformation results of lattice impeller and that of solid impeller is used to dimensionless and this ratio is defined as the deformation ratio. The deformation ratio reflects the deformation of lattice impeller relative to solid impeller and the results are shown in Fig. 2:

Compared with Fig. 2, we found that the average deformation of lattice impeller and unfilled impeller with lattice beam diameter of 0.2 mm, 0.4 mm and 1 mm was reduced by 4.84%, 3.49%, 3.71% and 7.31% respectively. This means that under the same inertia load, the actual aerodynamic performance of lattice impeller will be closer to the design value than that of solid impeller. This means that the lattice impeller will be closer to the design working range, and will not deviate from the design range. At the same time, due to the use of titanium alloy, even if there is a deviation from the speed, it still has a high safety reserve. Although the average circumferential deformation of the unfilled impeller is the smallest, its stiffness is difficult to meet the engineering requirements. As shown in Fig. 1a, the unfilled impeller has the largest radial deformation, and such large deformation is not easy to adjust for the unfilled impeller. In actual use, this will cause the reduction of shroud clearance, thus affecting the performance. Compared with the unfilled impeller, the lattice impeller has more excellent adjustability and flexibility. For path 1, no matter which direction of deformation, the deformation curve distribution of lattice impeller is located between the solid impeller and the unfilled impeller, and with the increase of lattice beam diameter, the deformation distribution of lattice impeller on path 1 is more and more close to the solid impeller, and when the lattice diameter decreases, the deformation distribution of lattice impeller is closer to the unfilled impeller.

The shape of blade will directly affect the aerodynamic performance of impeller. Under different working conditions, the blade shape of the impeller will be different, which means that there is the possibility that, while realizing the lightweight design of the impeller, it can adjust the deformation shape of the blade in the rotating state by changing the lattice beam diameter of the internal lattice structure, so that the aerodynamic performance of the blade can meet the engineering requirements, thus changing the service performance of the impeller under different working conditions. It will have great application prospect in turbojet engine and turbofan engine.

**Figure 3:**
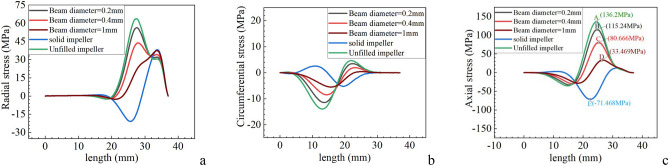
Stress of path 1|| (**a**) Radial stress; (**b**) Circumferential stress; (**c**) Axial stress.

The stress distribution of path 1 in three directions is shown in Fig. 3. From Fig. 3, it is found that for path 1, regardless of the stress in any direction, the distribution curve of lattice impeller is still between the unfilled impeller and the solid impeller. The axial stress with the largest stress in three directions is considered to evaluate the strength performance of several impellers. Figure 3c shows the axial stress of several impellers. The maximum stress of unfilled impeller and lattice impeller is tensile stress, and the maximum stress of solid impeller is compressive stress. Without filling impeller, the stress amplitude of lattice impeller and solid impeller with lattice beam diameter of 0.2 mm, 0.4 mm and 1 mm are respectively located at A, B, C, D and E positions in Fig. 3c, with values of 136.2 MPa, 115.24 MPa, 80 MPa, 33.469 MPa and − 71.468 MPa. Among them, the axial stress amplitude of unfilled impeller is the largest, so its strength is worse than that of lattice impeller and solid impeller.

**Figure 4:**
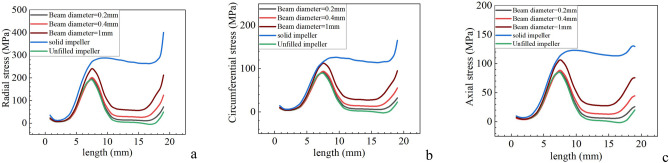
Stress of path 2|| (**a**) Radial stress; (**b**) Circumferential stress; (**c**) Axial stress.

Path 2 is located at the axis of the impeller hub, so the path basically does not produce deformation, so it is meaningless to study its deformation. However, the stress value at the impeller hub axle is large, which is the place where the impeller is prone to failure. Therefore, the stress distribution on Route 2 is mainly explored. The stress distribution of path 2 is shown in Fig. 4.

From Fig. 4, it can be found that the stress distribution curve of lattice impeller is still between the unfilled impeller and the solid impeller. In path 2, the stress curve still satisfies the law that the stress curve gradually approaches to the solid impeller with the increase of lattice filling rate. For the lattice impeller, due to the reduction of the internal mass of the impeller, the stress values in three directions of the impeller axle are greatly reduced. Therefore, the design of lattice structure is better than that of solid impeller. For high-speed impeller equipment, such as turbine compressor impeller, under the condition of emergency braking, it will cause less damage to the axle and reduce the braking time.

## Discussion

In this paper, a new lightweight compressor impeller based on the conventional cubic lattice is designed by using the rotational symmetry of the axisymmetric structure. Lattice filling method is used for the lightweight design of compressor impeller, under the inertia load only considering the limit speed of 80,000 r/min, the following conclusions are obtained through the finite element analysis. The following conclusions are reached based on the results:

1. We have redesigned the form of cubic lattice, which can satisfy the filling of axisymmetric structure and improve the stiffness and strength compared with the conventional lattice. The hub of the impeller is filled by the deformed cubic lattice structure, which greatly reduces the quality of the impeller. When the diameter of lattice bar is 0.4 mm, the mass of impeller can be reduced by 22.68% compared with the original design. The machinability of impeller is the premise of its actual production. We have successfully used SLM Solutions Group AG Germany metal 3D printer to verify the excellent machinability of the designed impeller.
2. Based on the good machinability of the impeller, the mechanical properties of the lattice impeller filled with different beam diameters are analyzed based on the finite element method. Through the finite element analysis, we found that the maximum deformation positions of the unfilled impeller, lattice impeller and solid impeller are all the outer edge positions of the large blade of the impeller. The lattice impeller and solid impeller have similar deformation distribution in the blade and hub, and the lattice structure in the lattice impeller hub is basically the same as that in the solid impeller hub. The stress distribution of blade is similar to that of lattice impeller and unfilled impeller. The von-Mises stress on the hub and blade of the lattice impeller with a lattice beam diameter of 0.4 mm is about 75 MPa smaller than that of the solid impeller and the unfilled impeller.
3. The average circumferential deformation of the lattice impeller is larger than that of the unfilled impeller and smaller than that of the solid impeller. This means that the lattice impeller will have more excellent aerodynamic performance than the solid impeller in actual operation, and because of its flexible adjustability and designability, it means that the lattice impeller will have better application prospects than the unfilled impeller and the solid impeller. The geometry of the lattice impeller blade can be adjusted by adjusting the diameter of the lattice beam, which provides a new possibility for the lattice impeller to meet certain operating conditions by controlling the internal lattice structure.

The actual operating conditions of the compressor impeller are very complex. In addition to its own "inertia" load, it also includes the static pressure on the impeller surface, the dynamic pressure due to the motion of the aircraft in the air, the thermal load and the mechanical load due to the vibration. In this paper, the design of lattice impeller is explored only considering the force of inertia load under high-speed rotation. In the further study, more complex conditions need to be considered. The impeller structure design also needs to further combine with the optimization algorithm to find a more reasonable material distribution form. In the next step, we will combine parameter optimization, topology optimization and other methods to achieve a more reasonable design of lattice impeller structure.

## Method

### Design of the model

ANSYS is used in the whole design and calculation process of disk model and impeller model. For the axis-symmetric structure, the rotating lattice has better radial tensile / compressive capacity than the conventional lattice in theory, and can resist the radial expansion deformation of the structure.

### Rotating lattice design

**Figure 5:**
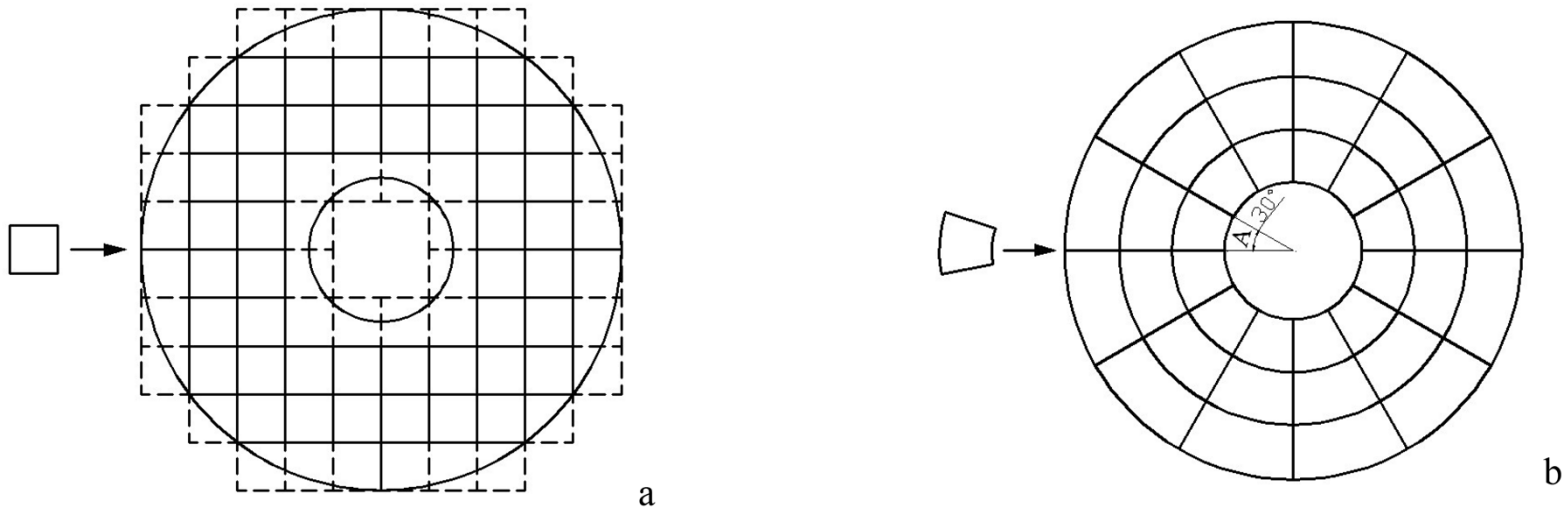
Comparison of two different filling methods|| (**a**) Conventional lattice filling; (**b**) “Rotation” lattice filling.

In the lightweight design of lattice structure, it is usually directly filled into the structure. Taking the regular cubic lattice as an example, if the lattice is directly filled into it, a large number of damaged lattice structures will be generated, as shown in the dotted line part of Fig. 5a. Due to the different broken areas, the stress in each part will be uneven, which will easily lead to the problem of local stress concentration. In order to solve this problem, using the rotational symmetry of the axis-symmetric structure, the conventional cubic lattice is transformed into a rotating lattice, and it is filled according to the arrangement shown in Fig. 5b to obtain the RL structure. For the RL structure, there is a minimum rotation angle, that is, the rotation angle of the rotation structure is the minimum rotation angle. For the structure shown in this paper, it is also equal to the angle corresponding to the minimum rotation array element. For the structure shown in Fig. 5b, the minimum rotation angle is angle A = 30°. This structure can ensure that every smallest array element (i.e. within a minimum rotation angle) has the same deformation shape.

### Performance comparison

In order to compare the force and deformation of two filling methods, i.e. rotating lattice and conventional lattice, taking the disk as an example, when only considering the inertia load of average rotation, the deformation and stress distribution are analyzed and compared. The conventional lattice structure and the RL structure are respectively filled in a disk (hereinafter referred to as the conventional lattice disk (CL disk) and the rotating lattice disk (RL disk) respectively). The same is that the filling ratio of the two kinds of disks is 25.97%, and the number of lattice cells is 480. In both cases, the profile of the CL disk enlarged by 10 times are shown in Figure S.1 (a), the profile of the RL disk is shown in Figure S.1b. Since the models in Figure S.1 (a) (b) are symmetrical around A-A and B-B planes, 1/4 of the models between A-B planes can be calculated according to the symmetry. The final design model of the two disks can refer to Supplementary Figure S.1.

The materials used for the two lattice disks are Ti6Al4V alloy commonly used in 3D printing industry, and the performance of the material at room temperature can refer to Supplement Table S.1. Ti6Al4V material is still used in the design of lattice impeller below.

The loading mode of disk model only considers the force and deformation under inertia load. The boundary conditions and meshing of RL disk can refer to Supplementary Figure S.2(a)(b), and the boundary conditions of conventional lattice are the same. Plane A constrains the circumferential displacement and applies a uniform rotational speed of 20,000 r/min around axis B. The finite element method is used to analyze the deformation and stress of two kinds of disks under specified working conditions. The volume mesh size is 0.2 mm, and the two disk models adopt the same volume mesh size. It is found that the relative error between the maximum deformation and stress amplitude is less than 1%, and the mesh quality of the two impellers can meet the calculation requirements.

**Figure 6:**
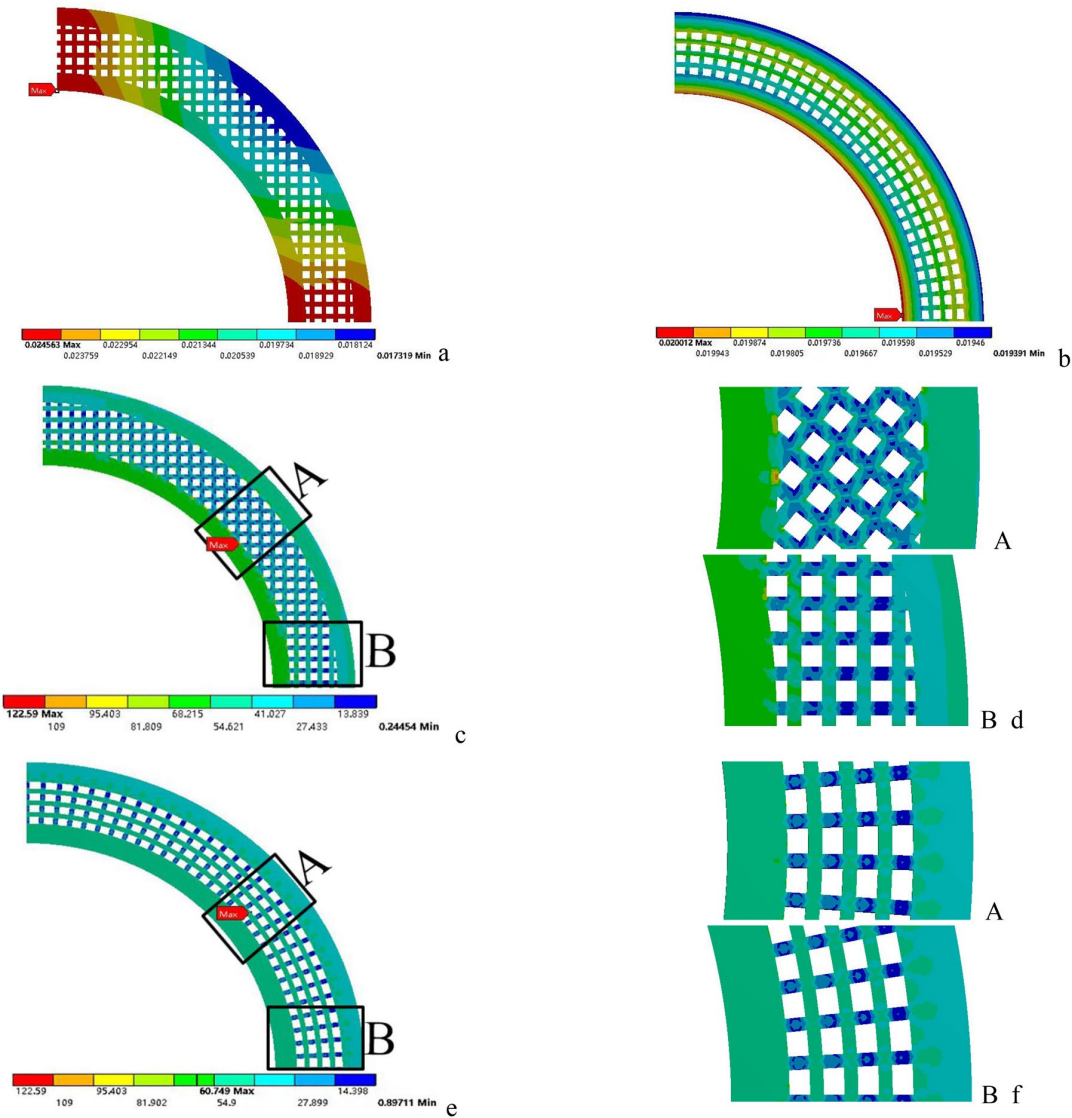
Stress and deformation distribution of two kinds of lattice discs|| (**a**) Deformation of CL disc; (**b**) Deformation of RL disc; (**c**) Stress of CL disc; (**d**) Amplification of parts with high stress; (**e**) Stress of RL disc; (**f**) Amplification of parts with high stress.

The deformation and stress distribution of the two kinds of lattice disks are shown in Fig. 6. The deformation unit is mm and the stress unit is MPa. Among them, Fig. 6a is the deformation of CL disk, Fig. 6b is the deformation of RL disk, and Fig. 6c,e is the stress of CL disk and RL disk respectively. For the convenience of comparison, the maximum value in the legend of Fig. 6e is adjusted to be consistent with Fig. 6c, and d, f is the partial enlarged drawing of stress distribution of two kinds of impeller discs. The maximum stresses and deformations of the two types of discs are shown in Fig. 6c,e.

It is found that under the above boundary conditions, the maximum deformation of the CL disk is 0.0246 mm, and the maximum stress is 122.59 MPa; The maximum deformation of the RL disk is 0.02 mm, and the maximum stress is 60.8 MPa. Among them, the maximum deformation and stress of RL disk are about 81.3% and 49.6% of that of CL disk, and the maximum deformation and stress of RL disk are obviously smaller than that of CL disk.

Compared with the deformation distribution in Fig. 6a,b, it can be found that the deformation distribution along the circumference of the RL disk is more uniform than that of the conventional lattice. This means that the stress concentration in the circular corner of the regular lattice disk is caused by the radial stretching. The stress distribution of the two discs is shown in Fig. 6c,e, and d,f is a partial enlarged view of A and B positions, A contains the maximum stress area of the whole structure, B is another area, and two kinds of disks intercept the same A and B. It can be found that the stress distribution at A and B of CL disk is quite different, and there is obvious stress concentration at A, as shown in Fig. 6d. However, the stress distribution in RL disk at A and B is basically the same, and the stress concentration is also weak, as shown in Fig. 6f. The stress distribution also shows that the RL disk has better strength than the CL disk.

It can be seen that for the RL disk, under the inertia load, it has obvious advantages over the conventional lattice filling. In the same way, when only considering the inertia load, the lattice impeller can also adopt this rotating lattice design method, so as to improve the performance of the impeller structure. This form of lattice structure design can also provide a reference for the lightweight design of other rotating structures.

### Structural design of rotating lattice compressor impeller

Taking a certain type of air compressor impeller as an example, some operating conditions of the impeller are shown in Supplementary Table S.2 and the geometric model of the original design impeller can refer to Supplementary Figure S.3. For the specific definition of angle in table S.2, please refer to references^21,22^.

The rotating lattice is applied to the lightweight design of compressor impeller, and the lattice compressor impeller is obtained. In the design of lattice impeller, the rotation stability of impeller and the force and machining performance of lattice impeller are all factors to be considered. For the internal lattice structure, a small minimum rotation angle can reduce the unbalanced mass force during the rotation of the impeller, improve the rotation stability of the impeller, and reduce the stress concentration of the lattice inside the lattice impeller. However, the minimum rotation angle cannot be set too small. Too small minimum rotation angle will make the lattice filling close to the impeller axle too tight, thus increasing the lattice filling rate and impeller mass. It will also make the powder materials that are not melted after printing not easy to flow out, affecting the use performance. Therefore, the design of minimum rotation angle needs to consider the above factors.

When 3D printing the internal lattice structure of impeller, the support cannot be removed after printing due to the support added to the internal lattice structure. Therefore, the printing process needs to ensure that the lattice beam can realize self-supporting without support, so that the lattice does not collapse when printing.

For Ti6Al4V alloy powder, when the lattice beam is connected at both ends, the maximum beam span L shall not exceed 1.5 mm. Leave a margin of 0.1 mm, and take the maximum L value of 1.4 mm to design the lattice. The single lattice cell can refer to Supplementary Figure S.4(a). Besides, the minimum array unit of the lattice inside the impeller can refer to Supplementary Figure S.4(b)., and its L has been marked in the figure.

**Figure 7:**
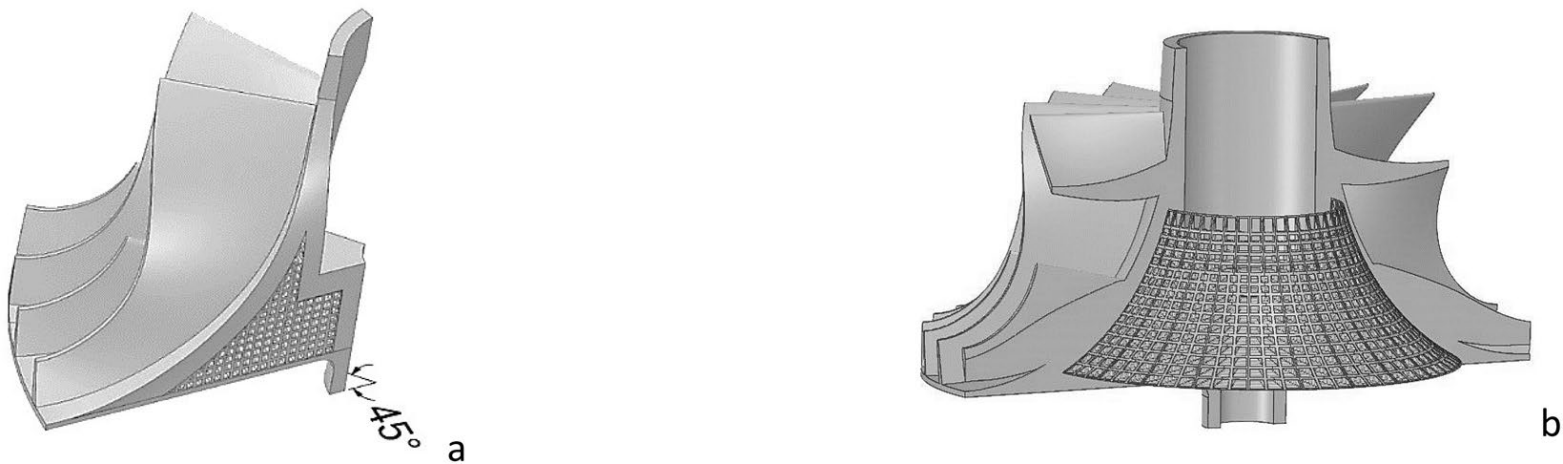
Correspondence between lattice and blades and lattice impeller model|| (**a**) Correspondence; (**b**) Model.

In order to minimize the unbalanced mass caused by the mismatching of the internal lattice structure and the distribution between the blades, it is better to achieve a one-to-one correspondence between each array unit of the internal lattice and the external blades in the design. Assuming that a large leaf and a small leaf are a group, there are 16 groups of leaves, each group of leaves corresponds to 22.5°. However, if the minimum rotation angle of the internal lattice is set to 22.5°, the L value will be too large and the internal lattice area cannot be printed. Based on the comprehensive consideration of the rotational symmetry of the lattice impeller and the machinability of the lattice area of the lattice impeller, the minimum rotation angle of the internal lattice is designed as 15°. In this case, every three minimum array units correspond to two groups of blades, as shown in Fig. 7a, that is, the minimum rotation angle of the whole lattice impeller structure changes to 45°.

The final lattice impeller design model is shown in Fig. 7b. The filling rate of lattice in lattice design domain is 21.5%, and the mass of lattice impeller is reduced by 22.68% compared with the original design model. In this case, the diameter of lattice beam is 0.4 mm. The lattice frame inside the impeller hub is a whole, which can be printed separately. The high speed balance is so important, in the conventional solid impeller, unbalanced mass can be removed from hub back disk. Similar to the traditional impeller, lattice impeller can also adjust the unbalanced mass through hub back disk. By changing the thickness of hub back disk to control the rigidity of impeller bottom, the filling area of lattice structure can be changed, but this may increase part of the mass to improve the rigidity of impeller bottom. We need to coordinate the pros and cons to maximize the benefits.

### Verification of machinability of lattice impeller

To ensure the machinability of the design structure is the premise of the practical application of the structure. In this section, SLM Solutions Group AG Germany metal 3D printer will be used to print the design model and test the machinability of lattice impeller.

The printing material is Ti6Al4V alloy powder commonly used in aerospace. The machining process of lattice impeller is divided into three stages: triangulation, slicing, printing and post-processing. By triangulating the solid model, generating the point cloud data of each part of the structure, and then slicing operation, the essence of slicing is the process of layered and discrete model, which makes the three-dimensional "body" into two-dimensional "face", so as to generate the print scanning path. The main parameter settings of the printing process can refer to Supplementary Table S.3.

**Figure 8:**
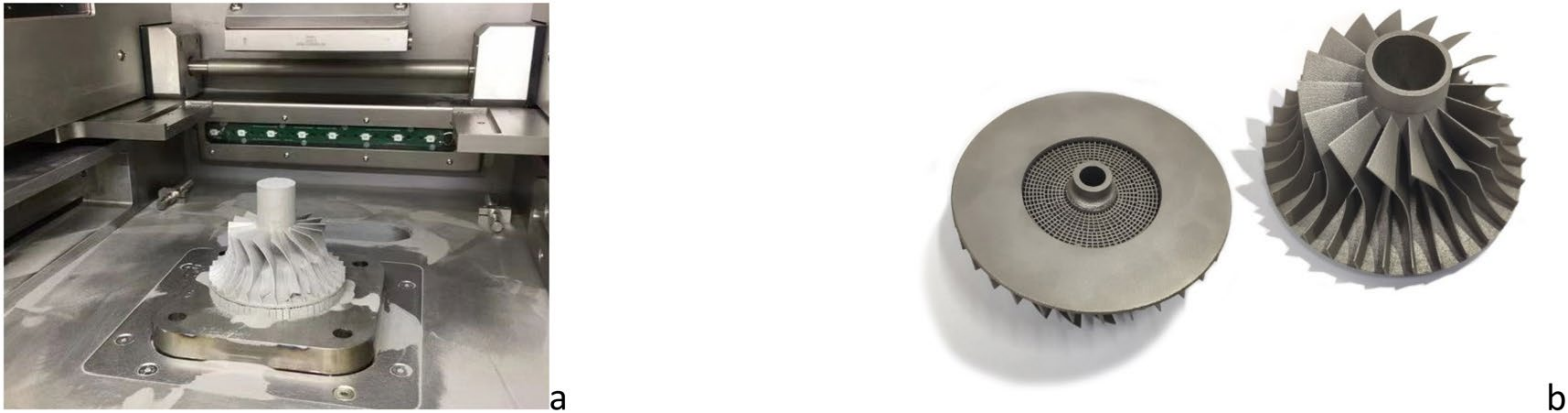
The unfinished lattice impeller (Support and base plate is not removed) and the finished lattice impeller || (**a**) The unfinished one; (**b**) The finished one.

During the printing process, there was no support fracture, visible crack and other faults on the parts, and the surface structure of the final product was complete. The lattice impeller when the support and base plate are not removed and the final model after printing is shown in Fig. 8a,b. There is no local fracture in the lattice structure inside the lattice impeller, and there is no printing failure in the lattice beam with large span. Therefore, it can be considered that the lattice impeller designed above is machinable. The surface of 3D printing impeller has a large surface roughness, but in the later stage, it needs to be milled once, so the efficiency will not be affected. On this basis, it is necessary to analyze some mechanical properties of the lattice impeller.

### Static performance under inertia load

The mechanical properties is one of the most important problems for impeller in the actual engineering design. For lattice impeller, its mechanical properties directly affect the aerodynamic performance and reliability of gas engine. In this section, three groups of lattice impellers with different lattice filling rate, solid impeller and unfilled impeller will be studied, and the stress and deformation distribution of three types of impellers at constant high speed will be compared and analyzed when only considering inertia load and the same boundary conditions.

### Analysis model simplification

Lattice structure model is more complex, so it is necessary to simplify the model properly in order to avoid consuming too much computing resources. In the calculation of lattice structure, the most commonly used simplified method is to convert lattice solid element to beam element for calculation^15^. Beam elements can resist bending, shear and torsional loads. It needs to define an accurate cross-section so that the program can calculate the moment of inertia, the neutral axis and the distance from the farthest element to the neutral axis, and its stress changes along the beam within the cross-section. The transformed beam model of lattice model and meshing can refer to Supplementary Figure S.5(a)(b).

### Grid independence analysis

In order to fully understand the deformation and strength change of the impeller only under its own mass force at high speed, the strength and deformation of the impeller are analyzed by finite element method without considering the surface pressure. Under the condition of "inertia" load and high speed of 80,000 r/min, the force and deformation of impeller are considered. The impeller adds the cylinder constraint (the coordinate is defined as the cylinder coordinate, where the X direction is radial, the Y direction is circumferential, and the Z direction is axial) at the position shown in Figure S.6(a), and adds the initial conditions of 80,000 r/min speed in the Z direction. Path 1 is on the outer edge of the large blade and path 2 is on the surface of the hub shaft hole. The boundary conditions and path selection of impeller can refer to Supplementary Figure S.6(a)(b).

In order to verify the rationality of grid setting, the radial stress of path 1 and path 2 and the radial deformation of path 1 are compared under different grid sizes, and the grid independence is verified. In impeller mesh generation, node tetrahedron mesh is used in hub, and node hexahedron mesh is generated by sweeping method in blade. The grid size of hub part is 1 mm, the grid size of blade part is 0.4 mm, and the number of divisions in the sweep method is set to 15. At this time, the grid is called coarse grid. Now change the number of grid and increase the grid density. The grid size of the grid hub is set to 0.5 mm, the grid size of the blade is set to 0.3 mm, and the number of strokes in the sweep method is set to 20. At this time, the grid is called fine grid. The coarse mesh and fine mesh can refer to Supplementary Figure S.7(a)(b).

The comparison results of the two grids on path 1 can refer to Supplementary Figure S.8. It can be seen from Figure S.8 that the simulation results of the two paths are basically the same, and the relative deviation between them is obviously less than 2%. Therefore, it can be considered that in this study, the finite element analysis with coarse mesh can meet the calculation requirements. In the next simulation settlement, coarse grid will be used for calculation to save computing resources and reduce simulation time.

### Finite element simulation

Blade and hub, lattice and hub are connected by bond. The blade and hub, lattice and hub connection type is "Bonded", and the formula is multipoint constraint (MPC), to define the coupling relationship between node degrees of freedom. The finite element analysis results of lattice impeller, unfilled impeller and solid impeller with beam diameter of 0.4 mm (deformation magnification of 120 times) can refer to Supplementary Figure S.9.

It can be seen from Figure S.9 (a) that the maximum deformation of lattice impeller mainly occurs at the outer edge of large blade and guide blade, and the closer to the shaft, the smaller the deformation. For the lattice structure inside the lattice impeller, the generated deformation also meets the law of gradually increasing along the radial direction from the shaft, and the internal lattice structure does not have local large deformation, as shown in Figure S.9 (b), and its deformation distribution is similar to that of the solid impeller in Figure S.9 (f). Compared with Figure S.9 (a) (c) (e), it can be found that the total deformation amplitudes of lattice impeller, unfilled impeller and solid impeller are about 0.053 mm, 0.062 mm and 0.041 mm respectively. The maximum total deformation of lattice impeller is larger than that of solid impeller and smaller than unfilled impeller, which is in line with the actual deformation distribution law.

Figure S.9 (g) shows the stress distribution outside the lattice impeller hub and between the blades, which is similar to Figure S.9 (i) stress distribution of the unfilled impeller. The maximum stress area of both hub and blade is at the hub back disk near the shaft hole, and the low stress distribution area is basically the same. Compared with Figure S.9 (i) (j) (l), it is found that the von-Mises stress amplitudes of lattice impeller, unfilled impeller and solid impeller are 290.38 MPa, 364.88 MPa and 367.64 MPa respectively. The stress amplitude of the unfilled impeller is similar to that of the solid impeller, while the stress amplitude of the lattice impeller is about 75 MPa lower than that of the unfilled impeller and the solid impeller at the blade and hub. For the parts printed with Ti6Al4V powder, the yield strength at room temperature is higher than 1000 MPa, so it can fully meet the engineering requirements.

The deformation shape and mechanical properties of the blade will directly affect the aerodynamic performance and reliability of the impeller. It is necessary to study the deformation and stress distribution of impeller more carefully.

## Supplementary information

Supplementary Information
