# Supplementary material for: Lightweight design and static analysis of lattice compressor impeller
Source: Sci Rep. 2020 Oct 27;10:18394. doi: 10.1038/s41598-020-75330-z (PMC7592040; doi:10.1038/s41598-020-75330-z)
Supplement: Supplementary file 1 — Supplementary Information [file 41598_2020_75330_MOESM1_ESM.docx]

Supplementary

Lightweight Design and Static Analysis of Lattice Compressor Impeller

Yuan ZHANG^1^, Fanchun LI^2,^ *, Dejun JIA^3^

^1,2,3^Dalian Maritime University, Dalian, Liaoning, 116026, China

| 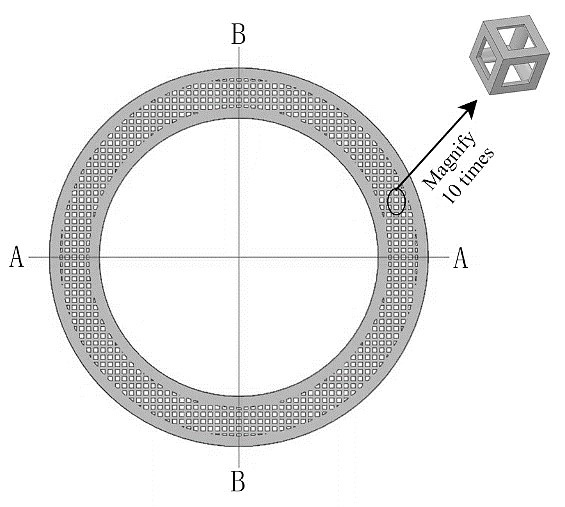 (a) | 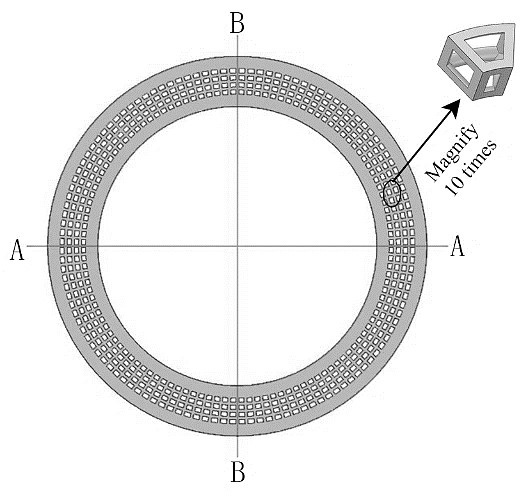 (b) |
| --- | --- |
| Figure S.1 Lattice pressure disc model\|\| (a) CL disc and (b) RL disc | |

| 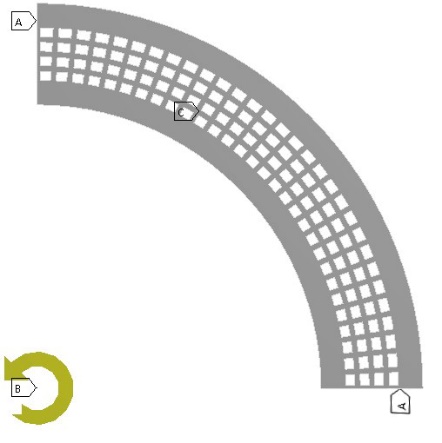 (a) | 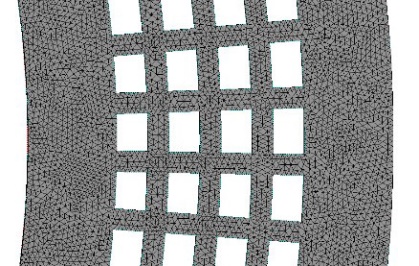 (b) |
| --- | --- |
| Figure S.2 Boundary conditions and meshing of disc\|\| (a) Boundary conditions (b) Meshing | |

| Table S.1 Properties of Ti6Al4V at Room Temperature | | | |
| --- | --- | --- | --- |
| Young modulus (MPa) | Poisson's ratio (-) | Yield strength (MPa) | Shear modulus (MPa) |
| 1.07×10^5^ | 0.323 | 1 098 | 4.04×10^4^ |

| Table S.2 Operation conditions of the impellers | | | |
| --- | --- | --- | --- |
| Overall pressure ratio | 4.5 | Tip clearance/Vane height | 0.02 |
| Rated speed | 80000 rpm | Hub diameter | 30mm |
| Vane normal thickness | 8 mm | Shroud diameter | 70mm |
| Inlet tip$\beta_{1}$ | 60° | Outlet tip$\beta_{2}$ | 45° |
| Main vanes/Intervanes | 16/16 |  |  |
| Rake angle | 30° |  |  |

| 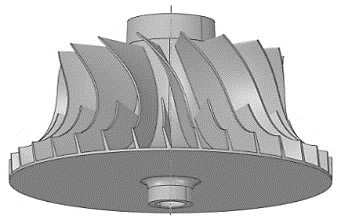 |
| --- |
| Figure S.3 The original model of the impeller |

| 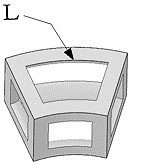 (a) | 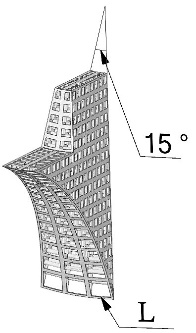 (b) |
| --- | --- |
| Figure S.4 Single lattice and minimum array unit\|\| (a) Single cell and (b) Minimum array unit | |

| Table S.3 Main parameters of impeller 3D-printing process | | | |
| --- | --- | --- | --- |
| Laser power (W) | 275 | Base plate temperature (°C) | 200 |
| Laser speed (m/s) | 1.1 | Initial deflection angle of laser (°) | 15 |
| Powder thickness (mm) | 0.03 | Layer deflection angle (°) | 67 |
| Scanning distance (mm) | 0.12 |  |  |

| 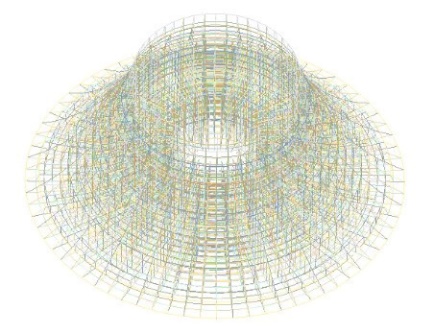 (a) | 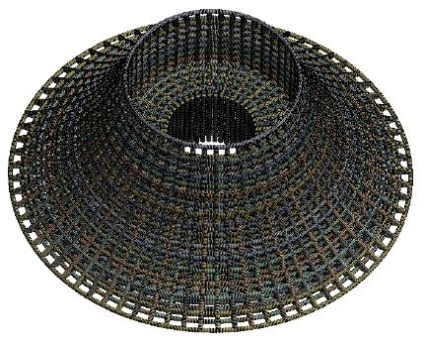 (b) |
| --- | --- |
| Figure S.5 Beam model and mesh generation of lattice beams\|\| (a) Beam model and (b) Mesh generation | |

| 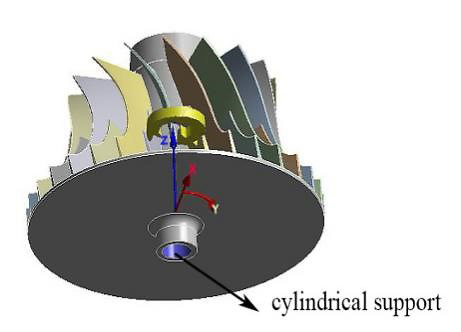 (a) | 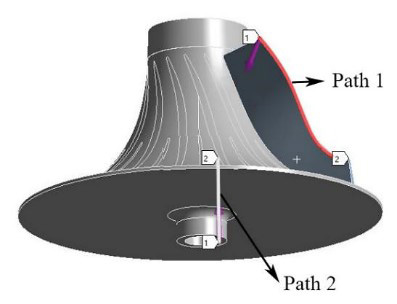 (b) |
| --- | --- |
| Figure S.6 Boundary conditions and path selection of impeller\|\| (a)Boundary condition and (b)Paths distribution | |

| 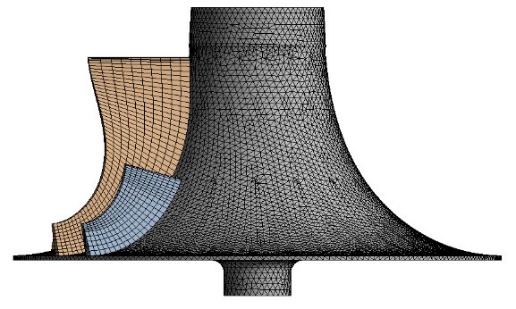 (a) | 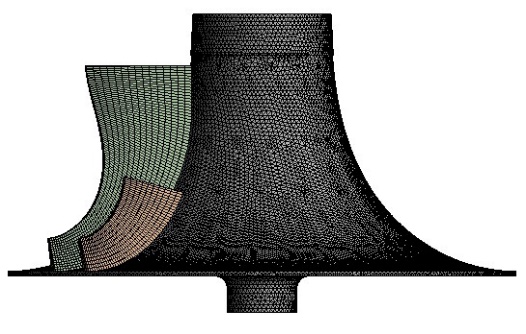 (b) |
| --- | --- |
| Figure S.7 Sparse grids and Fine grids\|\| (a)Sparse grids and (b)Fine grids | |

| 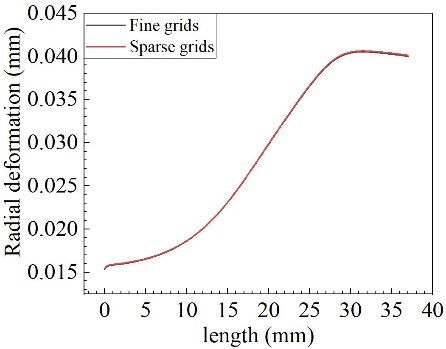 (a) | 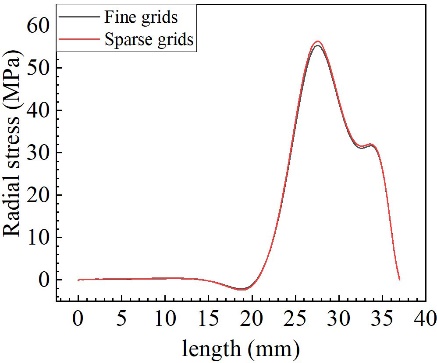 (b) | 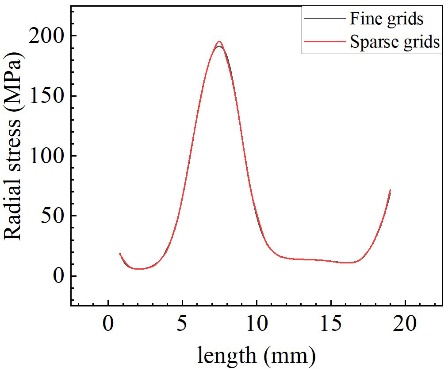 (c) |
| --- | --- | --- |
| Figure S.8 Comparison of results under different grid densities\| (a)Radial deformation of path 1; (b)Radial stress in path 1 and (c)Radial stress in path 2 | | |

| 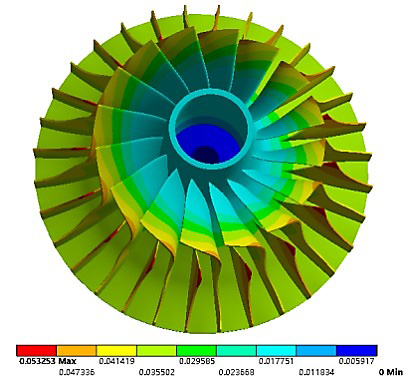 (a) | 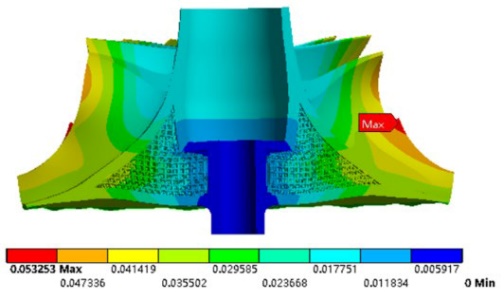  (b) | 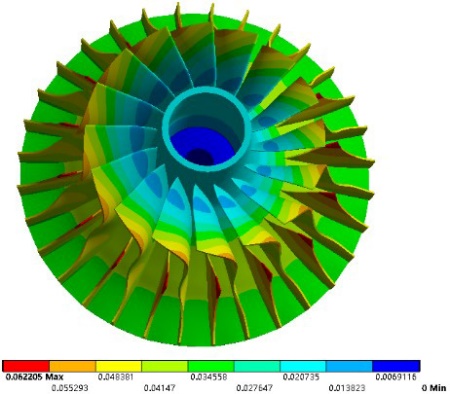 (c) |
| --- | --- | --- |
| 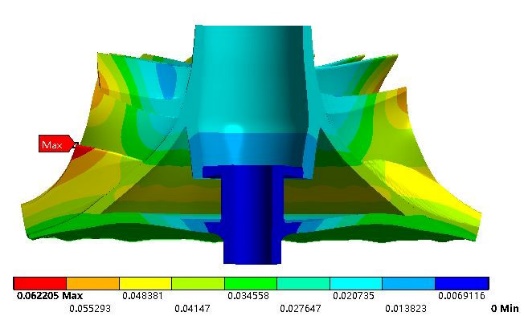  (d) | 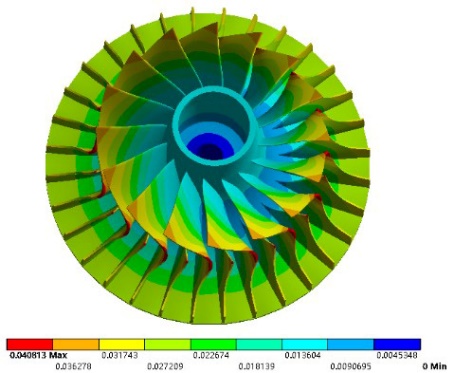 (e) | 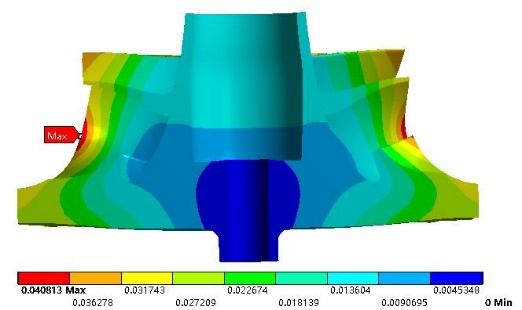  (f) |
| 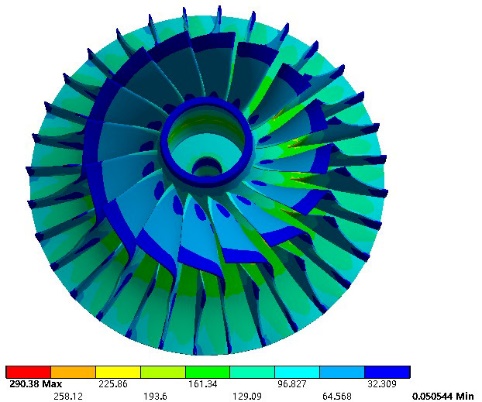 (g) | 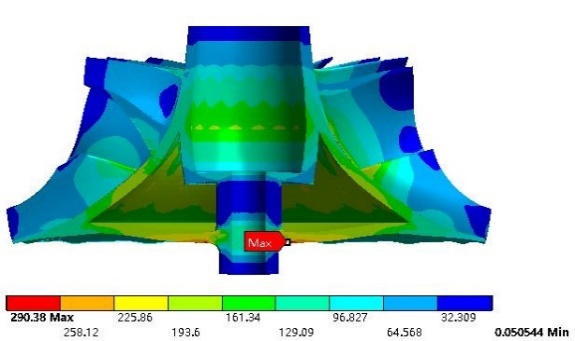  (h) | 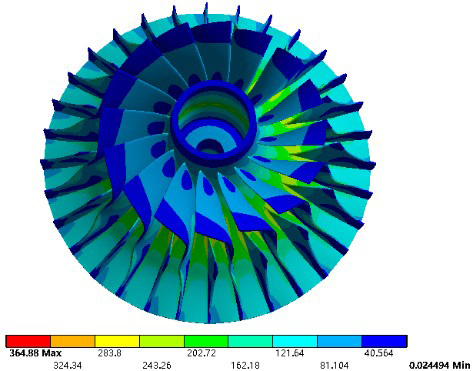 (i) |
|  |  |  |
| 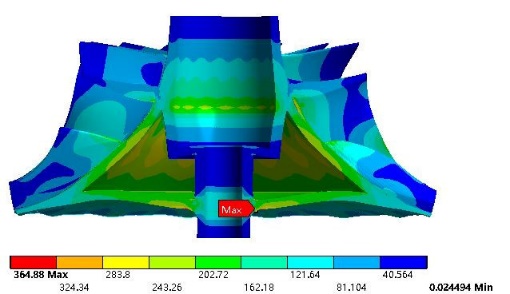  (j) | 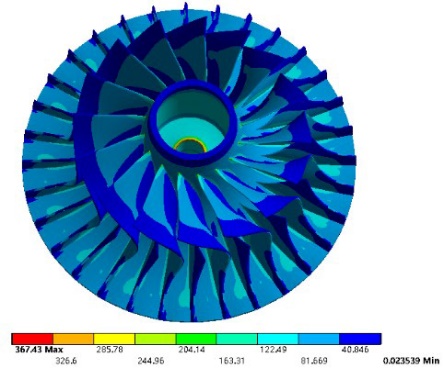 (k) | 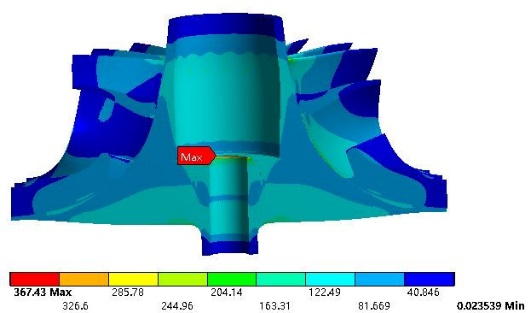  (l) |
| Figure S.9 Total deformation and von-Mises stress of lattice impeller (LTd and LVs), total deformation and Von-Mises stress of unfilled impeller (UTd and UVs) and solid impeller (STd and SVs)\| (a) LTd; (b) LTd (Section); (c) UTd; (d) UTd (Section); (e) STd; (f) STd (Section); (g) LVs; (h) LVs (Section); (i) UVs; (j) UVs (Section); (k) SVs and (l) SVs (Section) | | |
